# Time-trends and correlates of obesity in Czech adolescents in relation to family socioeconomic status over a 16-year study period (2002–2018)

**DOI:** 10.1186/s12889-020-8336-2

**Published:** 2020-02-13

**Authors:** Erik Sigmund, Dagmar Sigmundová, Petr Badura, Jaroslava Voráčová, Hobza Vladimír, Tomáš Hollein, Jan Pavelka, Zuzana Půžová, Michal Kalman

**Affiliations:** 1 Faculty of Physical Culture, Institute of Active Lifestyle, Palacký University Olomouc, 771 11 Olomouc, Czech Republic; 2 Faculty of Physical Culture, Department of Recreation and Leisure Studies, Palacký University Olomouc, 771 11 Olomouc, Czech Republic; 3 Faculty of Physical Culture, Department of Social Sciences in Kinanthropology, Palacký University Olomouc, 771 11 Olomouc, Czech Republic

**Keywords:** Obesity, Physical activity, Socioeconomic status, Health behaviour of school-aged children study

## Abstract

**Background:**

The main objective of the study is to analyse the changes in the prevalence of obesity among Czech adolescents between 2002 and 2018 with regard to the socioeconomic status (SES) of adolescents’ families and to find SES-separated correlates of adolescents’ obesity in 2018.

**Methods:**

A nationally representative sample of 29,879 adolescents (49.6% of them boys) aged 10.5–16.5 years was drawn from the Health Behaviour in School-aged Children cross-sectional, self-reported questionnaire surveys conducted in 2002, 2006, 2010, 2014, and 2018 in Czechia. Chi-square (*χ*^2^) tests were performed to assess the changes in the prevalence of obesity in both genders and all SES categories of adolescents between 2002 and 2018, and SES category-related differences in the prevalence of obesity in 2018 separately for boys and girls. A series of multiple stepwise logistic regression (backward elimination) analyses were used to reveal obesity correlates separately for SES categories of adolescents.

**Results:**

Across the quadrennial surveys from 2002 to 2018, we observed a clear increase in the prevalence of obesity in all SES categories of adolescents, which was most striking (*p* < 0.05) in adolescents with low SES (boys: + 7.5% points (p.p.); girls + 2.4 p.p.). When all the survey cycles were compared, the highest prevalence of obesity was evident in the low-SES adolescents in 2018, both in girls (5.1%) and boys (12.0%). Regardless of the adolescent SES category, the lower odds of obesity were significantly (p < 0.05) associated with regular vigorous physical activity (PA), participation in organized sport, and daily consumption of sweets. In addition, at least 60 min of moderate-to-vigorous PA significantly reduced the odds of obesity in adolescents of low and high SES categories.

**Conclusions:**

An unreasonable increase in the prevalence of obesity in adolescents with low SES highlights the need to prevent obesity in adolescents with a low-SES background. Additionally, significantly higher odds of obesity in 11- and 13-year-old adolescents from low-SES families, compared with their peers aged 15, indicated an expectable rise in obesity in older low-SES adolescents in the near future.

## Background

The epidemic of obesity in every segment of the population is a serious public health problem in the high-income as well as low- and middle-income countries of the world [1–3]. The prevalence of obesity is generally lower but is increasing faster in low- and middle-income countries than in high-income countries [4, 5], but the evidence from, for example, the former Communist Bloc countries in Europe is still limited [6]. On the evidence of the existing prevalence and trend data [1, 7–9] and the epidemiological evidence linking obesity with long-term cardiovascular, metabolic, and other health consequences [10], it is necessary to describe obesity as a public health crisis with serious negative impacts on the quality of life of people and imposing a considerable burden on national health-care budgets [11, 12].

To simplify, obesity is the result of a long-term positive energy balance between food intake and expenditure, with its rate being mediated by the complex interaction of multiple behavioural, biological, and environmental factors [13–16]. Lifestyle behaviour is strongly associated with obesity in school-aged children, regardless of country or continent [13, 15, 16]. Everyday physical activity of moderate-to-vigorous intensity (MVPA) and longer sleep duration have been associated with lower odds of overweight or obesity in school-aged children [16], while shorter sleep duration and longer outside-of-school screen time (ST – watching television and playing video/computer games) have been associated with higher odds of obesity [15, 16]. The consumption of sugar-sweetened beverages as an example of unhealthy food intake has been shown to be one of the key contributors to the risk of child overweight/obesity [17]. Although the consumption of sugar-sweetened beverages has declined over the last 15 years, it is still high among children and adolescents, with a negative impact on health – a higher incidence of obesity, insulin resistance, and dental caries [17]. Many of the energy balance-related behaviours in children and young people vary considerably with regard to the socio-economic status (SES) of their families [6, 16, 18, 19].

One of the major social determinants of child and adolescent obesity is the SES of their families [7, 9, 20]. Only a few of the trend-related publications that exist reveal a growing socioeconomic gradient in high-income countries – a stabilized or slight decreasing trend in the prevalence of obesity in children and adolescents from high-SES families, as opposed to a steady increase in the prevalence of obesity among their peers from low-SES families [7, 9,20]. Between 2003 and 2014, active participation in the Special Supplemental Nutrition Program for Women, Infants, and Children in Los Angeles [20] was first accompanied by an increase in childhood obesity (2003–2005), followed by a stagnation of obesity (2005–2010), and then a final decline in childhood obesity (2010–2014), with significant differences between children from different SES backgrounds. In most years, the incidence of childhood obesity was highest in the families with the lowest SES [20]. Bann et al. [21] point to a diametric change in the relationship between SES and body weight in a longitudinal survey. In the cohorts before 2001, lower SES was associated with lower weight and inequalities did not differ systematically with age until the 2001 cohort, in which weight and BMI inequalities widened at older ages. Nevertheless, critical information gaps persist in relation to the impact of childhood and life course SES on obesity in low- and middle-income countries [22], as well as an analysis of the impact of health-related programmes on the prevalence of obesity regarding SES [5].

Low- and middle-income European countries (including the countries of Central and Southern Europe from the former Communist bloc) appear to tend to replicate the ‘negative’ health-related behaviour patterns – a decrease in MVPA, an increase in ST [6, 23, 24] – which had previously been reported in high-income countries [25]. The rapid increase in childhood and adolescent obesity in low- and middle-income countries underlines the fact that these countries have failed to learn from the development of obesity in high-income countries. Czechia is one of the most economically developed European countries in the former Communist bloc [26]. Since 2006, it has been implementing a number of national health-related and sports programmes (such as “The Olympic Flag of Versatility”, “School Fruit and Vegetable Scheme “, and “School Milk Scheme”) for children and adolescents, supported by the Ministry of Education, Youth, and Sports [27]. However, the effect of these programmes on the health and health-related behaviour of children and adolescents is not monitored and evaluated to an adequate extent.

The present study attempts to bridge the gap between the national health-related and sports programmes that have been implemented and the lack of evidence of their effectiveness in terms of their potential impact on the prevalence of obesity. Therefore, the main objective of the study is to analyse the changes in the prevalence of obesity among Czech adolescents between 2002 and 2018 with regard to the SES of adolescents’ families and to find SES-separated correlates of adolescents’ obesity in 2018.

## Methods

### Study design

This study is based on five cycles of the Czech “Health Behaviour in School-aged Children” (HBSC) cross-sectional study. The HBSC study is a World Health Organization (WHO) collaborative cross-national study conducted in 48 countries across Europe, North America, and Asia. The HBSC study focuses on the description of adolescents’ health and health-related behaviours to inform policy makers, professionals, and practitioners to improve adolescents’ lifestyle [28]. An international standardized questionnaire and research protocol were used across all the participating countries to ensure the consistency of survey instruments, data collection, and processing procedures [28, 29]. As HBSC is a school-based survey, data is collected through self-completion questionnaires administered in the classroom. This study builds on methodology from a previously published work [18] and extends it by the most recent survey data and different analytical approach.

### Participants and data collection

Nationally representative samples of Czech adolescents in the age range 10.5–16.49 years were recruited during the 2002 (*n* = 4912; 47.7% boys), 2006 (*n* = 4629; 50.4% boys), 2010 (*n* = 4121; 48.7% boys), 2014 (*n* = 4588; 47.6% boys), and 2018 (*n* = 11,629; 50.4% boys) school years by using multistage stratified designs, with census regions, ratio of primary vs. multi-year grammar schools, and grades as strata, with schools acting as the primary sampling units (Table 1). The school response rate among the survey cycles varied from 75 to 99% and the adolescent participants’ response rate exceeded 80%. The participating teenagers were predominantly white Caucasian (> 96.5%), which corresponds to the very homogeneous ethnic demography of the Czech Republic [32].

**Table 1:** Descriptive characteristics of the samples, HBSC study, Czech Republic 2002–2018

	2002		2006		2010		2014		2018†	
	Boys	Girls	Boys	Girls	Boys	Girls	Boys	Girls	Boys	Girls
n=	(2345)	(2567)	(2332)	(2297)	(2008)	(2113)	(2183)	(2405)	(5856)	(5773)
	**%**	**%**	**%**	**%**	**%**	**%**	**%**	**%**	**%**	**%**
Age category^§^										
11 years	34.1	33.1	31.4	31.2	32.6	30.6	29.9	30.3	32.7	32.8
13 years	32.5	33.8	33.5	33.9	31.4	35.2	33.9	34.4	34.5	34.4
15 years	33.4	33.1	35.1	34.9	36.0	34.2	36.2	35.3	32.8	32.8
SES										
Low	34.2	40.5	25.7	30.3	13.1	17.0	32.4	34.4	23.7	27.2
Medium	56.5	53.0	54.9	55.9	54.2	53.3	44.3	45.4	45.3	44.6
High	9.3	6.5	19.4	13.8	32.7	29.7	23.3	20.2	31.0	28.2
Weight status*										
Non-overweight	81.6	91.7	77.9	83.8	73.7	88.1	74.8	87.9	73.3	84.7
95% CI	80.0–83.2	90.6–92.7	76.2–79.6	82.3–85.3	71.8–75.7	86.7–89.5	73.0–76.6	86.6–89.2	72.1–74.4	83.8–85.7
Overweight	14.5	6.5	14.2	11.8	18.7	9.5	18.1	9.3	17.9	11.9
95% CI	13.1–16.0	5.6–7.4	12.8–15.6	10.5–13.1	17.0–20.4	8.3–10.7	16.5–19.7	8.2–10.5	16.9–19.0	11.0–12.7
Obesity	3.9	1.8	7.9	4.4	7.6	2.4	7.1	2.8	8.8	3.4
95% CI	3.1–4.7	1.3–2.3	6.8–9.0	3.6–5.3	6.4–8.8	1.7–3.1	6.0–8.2	2.1–3.5	8.1–9.6	2.9–3.9

^§^11 years (13 years and 15 years) includes adolescents in the age range 10.5–12.49 years (12.50–14.49 years and 14.50–16.49 years); † in the 2018 data analyses, the weights for strata (administrative regions) were applied; *SES* socioeconomic status; *obesity and overweight were represented by the >97th percentile and 85th–97th percentile, respectively, on gender-specific Body Mass Index-for-age growth charts [30, 31]; *CI* 95% confidence interval

Trained researchers collected the data during a single morning lesson in the classroom using pen-and-paper questionnaires between 2002 and 2014 and in the IT classroom using online questionnaires in 2018. The participation of the adolescents in the quadrennial surveys was voluntary, and the respondents were assured of anonymity and the confidentiality of their responses. To ensure anonymity, the participants were instructed to put the questionnaire in an envelope, which they sealed and handed over to the researchers (2002–2014) after the survey was completed.

In 2018, during the online data collection, each participant received a unique application code to access a questionnaire from the researcher. The written consent of participants was obtained through the school management at all the schools involved in the survey. All the survey procedures for each data collection cycle were stored and can be downloaded from [33]. This study was approved by the Institutional Research Ethics Committee of the Faculty of Physical Culture, Palacký University Olomouc, with the reference No. 9/2016 on 4th March 2016.

### Survey items

#### Obesity

The adolescents’ self-reported actual body weight (kg), body height (cm), and chronological age were used to calculate the weight status of the participants. The Body Mass Index (BMI) was calculated as body weight (kg) divided by body height (m) squared. Body weight status (non-overweight, overweight, obesity) was determined in accordance with the WHO’s gender-specific BMI-for-age growth charts [31]. Obesity and overweight were represented by the >97th percentile and 85th–97th percentile, respectively, on the gender-specific BMI-for-age growth charts [30, 31] (Table 1). The BMI calculated from self-reported body height and weight demonstrated good diagnostic ability to identify excessive body weight in children and adolescents compared with direct anthropometric measurements (sensitivity, specificity, positive and negative predictive values ranged from 0.83 to 0.98) [34]. Self-reported body height and weight show high agreement with the values measured in the laboratory (Pearson correlation r_HEIGHT_ = 0.82–0.95 *p* < .001; r_WEIGHT_ = 0.90–0.96 *p* < .001), thus making it possible to identify excessive body weight in children and adolescents in epidemiological studies substantially [34–36].

#### Socioeconomic status

Because of repeatedly detected gender and socio-economically related differences in the prevalence of excessive body weight [18, 37] of adolescents, all the analyses were stratified according to the gender of the participants and family SES. An estimate of family SES was provided by the Family Affluence (FA) Scale. The FA scale comprised several simple-to-answer age-appropriate questions created to quantify material assets in the family [38, 39]. Between 2002 and 2010, four questions were included to determine FA: *having one’s own bedroom* (No = 0; Yes = 1), *number of computers* (None = 0; One = 1; Two = 2; Three or more = 3), *car ownership* (No = 0; One = 1; Two or more = 2), and *family holidays in the past year* (Never = 0; Once = 1; Twice = 2; Three or more times = 3) [38]. In 2014, an updated version of the FA scale was used to compensate for the changing social environment [23, 28]. Two new FA-related items – *dishwasher ownership* (No = 0; Yes = 1) and *number of bathrooms* (None = 0; One = 1; Two = 2; Three or more = 3) were added to the existing FA scale. The response codes to these items were summed and treated as a composite sum score. For the analyses, three categories of SES (“low”, “medium”, and “high”) were created from the composite sum score. Between 2002 and 2010 the SES categories correspond to tertiles of the sum score (“low” = 0–3; “medium” = 4–6, “high” = 7–9) and in 2014 and 2018 as follows: “low” = 0–6, “medium” = 7–9, and “high” = 10–13 [18]. High validity (kappa coefficient 0.41–0.74; 76.2–88.1 agreement) and moderate reliability (Cronbach’s α = 0.58) between children’s and parents’ responses on the FA scale-related items have been documented repeatedly [40–44]. Under the social and economic conditions of Czechia, the FA scale was validated with respect to the gross domestic product (Pearson correlation r = 0.773 *p* < .001) [45].

#### Energy balance-related behaviours

The energy balance-related behaviours covered physical activity patterns (daily MVPA, weekly vigorous physical activity (WVPA), and participation in organized sport), sedentary behaviour (daily entertainment screen time), dietary patterns (daily consumption of fruits, vegetables, sweets, and sweetened soft drinks; daily breakfast on school and weekend days, and frequency of fast food meals).

Among all the survey cycles MVPA was examined by a single question, ‘*Over the past seven days, on how many days were you physically active for a total of at least 60 minutes per day?*’ A definition of MVPA was provided as any activity that usually increases your heart rate and makes you get out of breath some of the time, with examples of activities that produce such an effect. The response categories were consistent among all the survey cycles and ranged from ‘*0 days’* to ‘*7 days’*. For the analyses of current MVPA recommendations (≥60 min per day [46]) a dichotomous outcome variable was created. The weekly participation in WVPA was determined by the question ‘*How often do you usually exercise in your free time so much that you get out of breath or sweat?’* with seven response categories: ‘*Every day’*, ‘*4 to 6 times a week’*, ‘*2 to 3 times a week’*, ‘*Once a week’*, ‘*Once a month’*, ‘*Less than once a month’*, and ‘*Never’*. In line with existing precedents [47], the outcome variable for WVPA was dichotomized as follows: ≥4 days of the week vs. less frequent WVPA. The assessment of self-reported MVPA and WVPA during the past seven days in adolescents was originally developed and validated against seven-day continuous measurement with an accelerometer (r_MVPA_ = 0.40 *p* < .01; r_WVPA_ = 0.36 *p* < .01) [48]. A recent study supports the validity of self-reported past-seven-days MVPA (r = 0.49 *p* < .01 correlation with seven-day continuous monitoring with an Actigraph accelerometer) [49], with almost perfect test-retest stability of the MVPA item in Polish (ICC = 0.98) and Chinese (ICC = 0.82) 11–15-year-old adolescents [50, 51]. The WVPA-related question in the HBSC questionnaire demonstrated moderate stability (ICC = 0.68) [51].

Participation in organized sport was investigated using the question on involvement in organized activities (six activities including team sports, individual sports): ‘*In your free time, do you do any of these organized activities? We mean activities you do in a sports or other club or organization*’ with the dichotomous response ‘yes*’*/‘no*’* [52]. The participating adolescents were categorized as *‘active’* (involved in organized team and/or individual sport) or *‘inactive’* (not involved in any organized sport). The scale of participation in organized sport has an acceptable level of agreement (ICC = 0.64), indicating good reliability [53].

In 2002, two items related to ST in free time were assessed. The adolescents were asked the following questions: ‘*About how many hours a day, in your free time, do you usually spend watching television (including DVDs and videos)?*’ and ‘*About how many hours a day, in your free time, do you usually spend using a computer (for playing games, emailing, chatting, or surfing on the internet)?*’ In all the other data collection cycles, the question related to computer use was subdivided into two questions to better reflect the changes in screen-based activities. The first sub-question, ‘*About how many hours a day, in your free time, do you usually spend using electronic devices such as computers, tablets (such as an iPad) or smartphones for other purposes, for example, homework, emailing, tweeting, Facebook, chatting, or surfing the internet?*’, represents the non-gaming part of computer use. The second sub-question, ‘*About how many hours a day, in your free time, do you usually spend playing games on a computer, games console, tablet (such as an iPad), smartphone, or other electronic device (not including moving or fitness games)?*’, represents the gaming part of computer use [54]. Each ST-related question was asked for weekdays and weekend days separately. For each ST-related question, the same response options were provided ‘*None at all’*, ‘*About half an hour a day’*, ‘*About 1 hour a day’*, ‘*About 2 hours a day’*, ‘*About 3 hours a day’,* ‘*About 4 hours a day’*, ‘*About 5 hours a day’,* ‘*About 6 hours a day’,* and ‘*About 7 or more hours a day’*. The validity of self-reported ST-related questions for the past seven days has been proved in comparison with 7-day 24-h diaries both on weekdays (r = 0.39–0.46, *p* < .001) and at weekends (r = 0.37–0.47, *p* < .001) [55, 56]. At least 7-day test-retest stability of the ST-related questions (computer use (PC) and television viewing (TV)) have been repeatedly verified among adolescents aged 11–15 for weekdays (ICC_PC_ = 0.33–0.82, ICC_TV_ = 0.54–0.72) and weekends (ICC_PC_ = 0.33–0.66, ICC_TV_ = 0.58–0.68) [50, 51, 55, 57]. The daily ST outcome variable comprised the sum of three (two in 2002) ST-related questions. In accordance with previous ST-related studies [58, 59], the cutoff point for the dichotomization of daily ST was set at *‘2 or more hours daily’* vs. *‘less than 2 hours daily’*. As part of the validated brief food frequency questionnaire, adolescents were asked: ‘*How many times a week do you consume fruit/vegetables/sweetened soft drinks/sweets?*’, with seven response categories: ‘*Never’*, ‘*Less than once a week’*, ‘*Once a week’,* ‘*Two to four times a week’*, ‘*Five to six times a week’*, ‘*Once a day’,* and ‘*More than once a day’* and ‘*How often do you usually have breakfast (more than a glass of milk or fruit juice)?*’ with six response categories for weekdays (‘*Never’*, ‘*One day’*, ‘*Two days’*, ‘*Three days’*, ‘*Four days’* and ‘*Five days’*), and three for weekends (‘*Never’*, ‘*Only on one day’*, and ‘*On both days’*) [19]. In line with previous trend-related studies [19, 60] the response options were recorded into dichotomous outcome variables, *‘daily’* vs. *‘less than daily’*. The last question investigated the frequency of eating in fast food restaurants: ‘*How many times a month do you eat in fast food restaurants?*’, with seven response categories: ‘*Never’*, ‘*Rarely’*, ‘*Once a month’,* ‘*Two to three times a month’*, ‘*Once a week’*, ‘*Two to four days a week’*, and ‘*Five days a week or more’*. The outcome variable for eating in fast food restaurants was dichotomized as follows: *‘at least twice a month’* vs. *‘less often’*.

#### Sleep time

Sleep time was calculated from the participants’ reports of bedtimes and wake-up times separately for school days and weekends. Bedtimes were asked about by a single question, ‘*When do you usually go to bed if the next morning is a school day (weekend day)?*’ Self-reported alternatives for bedtimes ranged at half-hour intervals from ‘*at least 9 pm’* to ‘*2 am or later’* for school days and ‘*at least 9 pm’* to ‘*4 am or later’* for weekend days. Wake-up times were asked about as follows: ‘*When do you usually wake up on school (weekend) mornings?*’ The response categories ranged at half-hour intervals from ‘*5 am at the latest’* to ‘*8 am or later’* for school days and ‘*7 am at the latest’* to ‘*2 pm or later’* for weekends [61]. Finally, sleep time was calculated as the difference between bedtime and wake-up time, separately for weekdays and weekend days. The outcome variable for sleep time was categorized in relation to the age-related recommendation for hours of sleep [62] as follows: *‘enough sleep’* vs. *‘not enough sleep’* for school days and *‘enough sleep’*, *‘not enough sleep’*, and *‘excessive sleep’* for weekends. The sleep-related items demonstrated at least substantial reliability (ICC = 0.75/0.64 for the usual bedtime on school days/weekends; ICC = 0.77 for wake up on school days), especially for the item on when the participants wake up at weekends, for which the reliability is almost perfect (ICC = 0.83) [51].

### Data analysis

All data processing and statistical analyses were performed in the Statistical Package for the Social Sciences (SPSS) for Windows v.22 software (IBM Corp. Released 2013. Armonk, NY, USA). Descriptive data are presented as percentages, including 95% confidence intervals (CI) in the case of weight status, for each survey cycle and gender separately. Chi-square (*χ*^2^) tests were performed to assess trend-related differences in the prevalence of overweight/obesity in each gender and SES categories of adolescents between 2002 and 2018, and SES category-related differences in the prevalence of overweight/obesity in 2018 separately for boys and girls. The chi-square (*χ*^2^) tests were also used to analyse the differences in the proportion of participants involved in organized sports and engaging in WVPA in relation to SES categories of adolescents and to test the statistical significance of differences in the prevalence of obesity by adolescents’ WVPA level, participation in organized sport, and frequency of consumption of sweets. A series of multiple stepwise logistic regression (backward elimination) analyses in the 2018 data collection were used to reveal obesity correlates separately for SES categories of adolescents. Finally, only statistically significant correlates of adolescent obesity will be presented. Given the larger sample, which represents the whole country and individual administrative regions, strata-specific weights were used for the 2018 data. The regression parameters were based on the odds ratio (OR) with a 95% CI. An alpha level of 5% was set for all the statistical procedures.

## Results

### Changes in prevalence of obesity

**Fig. 1:**
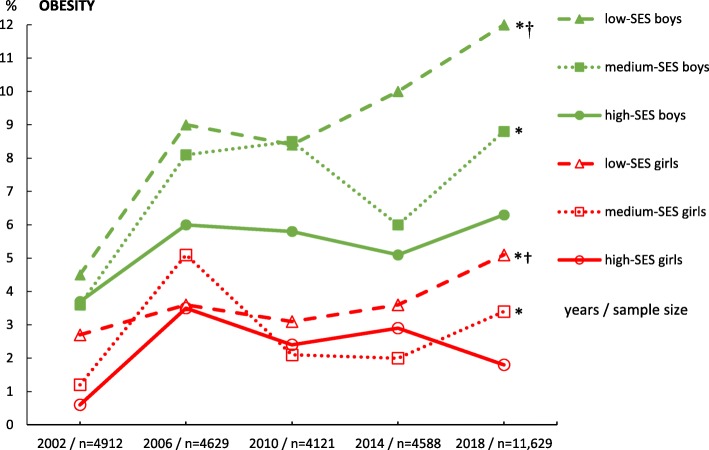
The changes in the prevalence of obesity in a randomized sample of Czech adolescents aged 11–15 years between 2002 and 2018. *N* Number, *SES* Socioeconomic status, *chi-square tests differences (*p* < .05) in the prevalence of obesity between 2002 and 2018, †*chi-square tests* differences (*p* < .05) in the prevalence of obesity between low- and high-SES categories of boys and girls separately in 2018

There is an evident increasing trend in the prevalence of obesity (Fig. 1) in adolescents of both genders from low-SES backgrounds between 2002 and 2018. A detailed overview of the prevalence of obesity (%) in girls and boys in each SES category in each year of data collection, including confidence intervals, is presented in Table 2.

**Table 2:** Prevalence of obesity‡ (%, (95% CI)) in adolescents by gender and SES between 2002 and 2018, HBSC study, Czechia

**Obesity**						
	**Year**	**2002**	**2006**	**2010**	**2014**	**2018**
**Boys**						
Low SES		4.5 (3.0-6.0)	9.0 (6.7-11.3)	8.4 (5.0-11.8)	10.0 (7.8-12.2)	12.0 (10.2-13.9)
Medium SES		3.6 (2.6-4.6)	8.1 (6.6-9.6)	8.5 (6.8-10.2)	6.0 (4.5-7.5)	8.8 (7.7-10.0)
High SES		3.7 (1.2-6.2)	6.0 (3.8-8.2)	5.8 (4.0-7.6)	5.1 (3.2-7.0)	6.3 (4.6-8.0)
**Girls**						
Low SES		2.7 (1.7-3.7)	3.6 (2.2-5.0)	3.1 (1.3-2.9)	3.6 (2.4-4.8)	5.1 (4.0-6.3)
Medium SES		1.2 (0.04-2.0)	5.1 (3.9-6.3)	2.1 (1.3-2.9)	2.0 (1.2-2.8)	3.4 (2.7-4.1)
High SES		0.6 (0.01-1.1)	3.5 (1.5-5.5)	2.4 (1.2-3.6)	2.9 (1.4-4.4)	1.8 (1.1-2.4)

*SES* socioeconomic status, *CI* 95% confidence interval

A significant increase in obesity between 2002 and 2018 was also uncovered in adolescents in the medium-SES category (Fig. 1). The ‘up-stairs’ effect in the prevalence of obesity (i.e. the sharp increase between 2002 and 2006 was replaced by stagnation/decline between 2006 and 2014, with a subsequent increase between 2014 and 2018) in the 2002–2018 period was typical for all SES categories of adolescents except for girls from the high-SES category. In addition, between 2014 and 2018, there was apparently not only an increase in the prevalence of obesity but also a widening of the difference in the prevalence of obesity between the low and high adolescent SES categories (Fig. 1).

### Correlates of obesity

Given the significant differences in the prevalence of obesity between the low- and high-SES categories of adolescents in 2018, obesity correlates are presented separately for all SES categories (Table 3). In all the cycles of data collection, girls in all SES categories reported having a lower prevalence of obesity than boys. Regardless of the adolescent SES category, the lower odds of obesity were significantly (*p* < .05) associated with regular WVPA, participation in organized sport, and daily consumption of sweets (Table 3). However, the lowest proportion of participants involved in organized sports (*p* < .001) and regularly engaging in WVPA is among the low-SES adolescents (*p* < .001).

**Table 3:** Correlates^#^ of obesity in randomized sample of Czech adolescents in relation to SES: HBSC survey in 2018

					Obesity				
		Low	SES		Medium	SES		High	SES
			95% CI			95% CI			95% CI
	%^a^	OR	lower-upper	%^a^	OR	lower-upper	%^a^	OR	lower-upper
Gender									
Girls	5.1	Ref.		3.4	Ref.		1.8	Ref.	
Boys	12.0	**2.11**^**‡**^	**1.70–2.61**	8.8	**2.29**^**‡**^	**1.93–2.72**	6.3	**2.39**^**‡**^	**1.89–3.03**
Age Category									
15 years	8.7	Ref.		5.5	Ref.		3.3	Ref.	
13 years	7.2	1.38*	**1.07–1.76**	6.7	**1.35**^**†**^	**1.11–1.63**	3.8	1.20	0.93–1.55
11 years	9.0	1.39*	**1.06–1.81**	6.2	1.17	0.95–1.46	5.4	1.18	0.88–1.58
60 min of MVPA									
0–6 days	8.8	Ref.		6.2	Ref.		4.7	Ref.	
7 days	5.5	0.67*	**0.47–0.96**	5.8	0.98	0.77–1.24	2.1	**0.63**^**†**^	**0.46–0.86**
Weekly vigorous PA									
< 4 times a week	9.1	Ref.		6.7	Ref.		4.6	Ref.	
≥ 4 times a week	6.7	0.73*	**0.57–0.93**	5.3	**0.76**^**†**^	**0.63–0.91**	3.6	0.78*	**0.62–0.99**
Participation in sport									
Inactive (no participation)	9.9	Ref.		7.4	Ref.		5.6	Ref.	
Team and/or individual	7.0	**0.66**^**‡**^	**0.53–0.81**	4.8	**0.69**^**‡**^	**0.58–0.82**	3.5	0.75*	**0.58–0.96**
Screen time on weekdays									
≥ 2 h per weekday	8.6	Ref.		6.5	Ref.		4.3	Ref.	
< 2 h per weekday	4.9	0.91	0.59–1.39	2.9	**0.47**^**‡**^	**0.32–0.68**	2.0	0.70	0.43–1.12
Breakfast on weekdays									
less than daily	8.8	Ref.		7.4	Ref.		4.3	Ref.	
daily	7.6	0.84	0.68–1.05	5.1	0.81*	**0.68–0.96**	3.9	1.07	0.85–1.35
Consumption of sweets									
less than daily	8.9	Ref.		6.5	Ref.		4.6	Ref.	
daily	5.4	**0.58**^**‡**^	**0.44–0.78**	4.1	**0.53**^**‡**^	**0.42–0.67**	2.0	**0.58**^**‡**^	**0.42–0.79**
Eating fast food									
less often	8.4	Ref.		6.3	Ref.		4.3	Ref.	
at least twice a month	8.0	0.81	0.60–1.09	5.3	0.77*	**0.61–0.97**	3.3	**0.57**^**‡**^	**0.43–0.77**
Sleep time at weekends									
enough sleep	7.9	Ref.		5.9	Ref.		4.4	Ref.	
not enough sleep	14.0	1.15	0.87–1.53	8.9	1.33*	**1.05–1.69**	5.5	1.48*	**1.09–2.00**
too much sleep	6.0	0.79	0.61–1.03	4.8	0.82	0.67–1.01	3.0	0.89	0.67–1.17

^#^significant in at least one of the adolescent SES categories, *SES* Socioeconomic status, *CI* 95% confidence interval, *%*^*a*^ percentage of obese adolescents per independent variable stratified by SES, *OR* odds ratio (logistic regression) of being obese in SES separately conducted analysis, *Ref.* Reference group, *PA* physical activity, *MVPA* moderate-to-vigorous physical activity, *p < .05, ^†^*p* < .005, ^‡^*p* < .001 Significant associations (*p* < .05) are in bold

Additional analysis confirms a significantly lower prevalence of obesity in adolescents with regular participation in WVPA compared with those with less frequent participation (5.0% vs. 6.8%, *p* < .001), active participants in sports compared with non-participating adolescents (5.0% vs. 8.1%, *p* < .001), and daily consumers of sweets compared with those who consume sweets less often (3.8% vs. 6.6, *p* < .001).

At least 60 min of MVPA daily generally reduce the odds of obesity in adolescents, but the finding was only significant in adolescents from low-SES and high-SES categories. Paradoxically, eating in fast food restaurants more frequently is associated with lower odds of obesity in adolescents (significantly in adolescents from the medium- and high-SES categories) (Table 3).

In the cohort of 11-year-old adolescents, we found the highest prevalence of obesity compared to 13- and 15-year-old adolescents (11 yrs. – 6.8%, 13 yrs. – 5.9%, and 15 yrs. – 5.6%), with the difference being significant between the cohorts of 11- and 15-year-old adolescents (*p* < .05). In adolescents with at least two hours of ST daily on school/weekend days we revealed a significantly higher prevalence of obesity than in adolescents with less than two hours of ST per day (6.4%/6.3% vs. 3.4%/3.5% *p* < .001). However, in the case of the SES categorization of adolescents, non-excessive ST at weekends is significantly associated with lower odds of the occurrence of obesity only in adolescents from the medium-SES category (Table 3). In adolescents who reported not getting enough sleep on school/weekend days, we found a significantly higher prevalence of obesity than among adolescents meeting the sleep recommendation (5.5%/5.9% vs. 6.9%/9.5% *p* < .01). A lack of sleep at weekends was significantly associated with higher odds of obesity in adolescents from the medium- and high-SES categories.

## Discussion

The key findings of the trend analysis between 2002 and 2018 include the revelation of an ‘up-stairs’ effect in the prevalence of obesity in all adolescents except for girls from the high-SES category and increasing differences in the prevalence of obesity between gender-separated low- and high-SES categories of adolescents.

Czechia has undergone rapid economic development and is one of the most economically developed countries in Central and Eastern Europe [26, 45]. This rapid economic development was reflected in the growth of families with high SES after 2010.

However, as in high- economic developed countries, rapid economic development is not always accompanied by positive health development of adolescents in all SES categories. It turns out that Czechia is repeating a similar development in child and adolescent obesity to economically more advanced countries (e.g. Australia, England, France, Germany, Netherlands, and the USA) 10–15 years ago, where the increase in obesity reached a plateau, with a subsequent increase in obesity among low-SES adolescents [7, 63].

After a sharp increase in obesity among Czech adolescents between 2002 and 2006, a number of national health-related and sports programmes (such as “The Olympic Flag of Versatility”, “Fruit and Vegetables in Schools”, and “Milk to Schools”) were introduced for children and adolescents with support from the Ministry of Education, Youth, and Sports [27]. A new compulsory subject called “Health Education” was also established at primary schools. This course focuses on healthy eating habits, non-risky behaviour (avoidance of drug use and smoking) and nature and environmental friendliness. These health-promoting activities may have contributed to the stabilization (plateau) of the prevalence of overweight/obesity among adolescents between 2006 and 2014.

However, following the reduction of nationwide financial support for some national programmes between 2013 and 2015 due to different government priorities, a rebound of overweight/obesity among adolescents was registered in the 2018 national data collection. The subsequent increase in overweight/obesity is most pronounced in adolescents with a low-SES background. The possible subsequent effect of national health and sport programmes is most noticeable in adolescents from high-SES families. In addition, significantly higher odds of obesity in the age categories of 11 and 13 years from low-SES families than among 15-year-olds indicated an expected rise in obesity in older low-SES adolescents in the near future. The trend patterns of excessive body weight, especially obesity in adolescents with a low-SES background and in the youngest age category examined between 2002 and 2018, indicate an urgent need for improvement. An international comparison of obesity changes between 2002 and 2014 across 27 European countries revealed that most market-driven obesity in Eastern European countries, where the levels of obesity were relatively low in 2002 and in adolescents with a low-SES background (6).

Special attention is therefore paid to the subsequent analyses of obesity correlates in adolescents from various SES backgrounds. Despite the differences in SES, three correlates of energy balance-related behaviours were identified as being associated with significantly lower rates of obesity in all SES groups of adolescents: i) regular WVPA (≥4 times a week), ii) active participation in sport, and iii) daily consumption of sweets. Moreover, in adolescents from low- and high-SES families, engagement in MVPA for at least 60 min a day also significantly reduces the risk of obesity. In adolescence, behaviour associated with more pronounced energy expenditure (PA of at least moderate intensity, participation in sport) appears to have a stronger anti-obesity effect than the absence of unhealthy eating habits. However, the energy expenditure required for adolescents must also include the energy required for bodily growth and development. Unlike other studies [64–66], more frequent eating (at least twice a month) in fast food restaurants in Czech adolescents with medium and high SES was associated with a significantly lower risk of obesity than in adolescents with lower rates of eating in fast food restaurants. On the other hand, regular breakfast is, in line with Marlatt et al. [66], associated with lower rates of obesity. These eating patterns are “more typical” for adolescents who regularly participate in sports than for non-participating adolescents. The participation of 11–15-year-old adolescents in sport was related to more frequent eating at fast food restaurants but less frequent snacking in front of the computer and intake of crisps than in non-sporting participants [67]. In addition, in the context of TV, it has been found that adolescents who watched TV for a longer time were more likely to consume sweets and soft drinks daily and less likely to consume fruit and vegetables [56]. However, a more significant obesity-related problem can occur when an adolescent ceases to participate in sports or is not regularly involved in MVPA and does not change his or her eating habits, because unhealthy eating habits adopted in adolescence tend to persist into adulthood and represent a crucial factor in the development of obesity [68–70].

Another explanation for the results that the daily consumption of sweets and eating more frequently in fast food restaurants are related to a lower likelihood of the prevalence of obesity is that non-obese adolescents do not have to care about unhealthy eating habits as much as their obese classmates, as evidenced by previous findings [71, 72]. Better eating habits in the obese category may have been influenced by the “social desirability” factor in addressing dietary habits in the HBSC questionnaire [73]. Undervaluation of responses to unhealthy and socially undesirable foods has proved to be commonplace in questionnaire surveys of overweight and obese participants [74]. The results could also be influenced by the current tendency among overweight or obese adolescents to reduce weight [75]. Because HBSC is cross sectional it is plausible that obese adolescents may be engaging in dietary restriction/healthier dietary habits at the time of the survey.

Excessive body weight is not only associated with long-term cardiovascular and metabolic health complications [10], but also with social and psychosomatic complications [71, 76]. Overweight/obese 11-to-15-year-old girls spend less time with friends after school, and overweight/obese boys report less frequent e-communication compared to normal-weight adolescents. In addition, the overweight/obese weight status of adolescents is associated with not perceiving a best friend as a confidant [76]. This finding is perhaps also one of the reasons why there are more individuals with normal body weight among the participants in organized team or individual sports. In addition, adolescents from low-SES backgrounds have been significantly more likely to fall behind their peers in terms of life satisfaction [71]. In addition to the financial and logistical demands of adolescents’ participation in organized leisure-time sport, this finding may contribute to explaining why the lowest proportion of participants in organized sport and regularly engaging in WVPA is among adolescents with low SES.

Although the lowest proportion of participants in sports and regular WVPA implementation is among the low-SES adolescents, at least 60 min of any MVPA daily can assist in reducing obesity. Therefore, in addition to sport, it is necessary to support and create the conditions for daily implementation of MVPA in all children and adolescents, regardless of the SES category. Improving public open spaces in low-SES areas by installing play spaces for recreational PA [77] or expanding school-related PA (including active recess, physical education lessons [78, 79] and after-school nursery [80]) has an impact on increasing day-to-day PA [80] and reducing children’s obesity [80, 81].

Short sleep duration is generally associated with increased obesity in European adolescents [82] as well as in adolescents from Canada and the United States [83, 84]. In addition, a positive relationship between shorter sleep duration and obesity appears to be related to both sides of energy balance-related behaviours as a result of a combination of increased food intake and more sedentary habits [82]. However, in our study, short sleep duration at weekends is associated with a significantly higher risk of obesity only in adolescents from medium and high SES backgrounds. A higher prevalence of obesity also appears to be related to the environments that children and adolescents reside in and in the neighbourhood of the schools they attend [65]. Fast food restaurants are more frequently present in low-SES neighbourhoods [19, 65]. The availability of fast food restaurants near the place of residence or schools is associated with lower consumption of fruits and vegetables, higher consumption of soft drinks, and increased odds of childhood obesity being diagnosed [65].

### Strengths and limitations

The large sample size, with high response rates in all the survey cycles, strict adherence to the international standardized questionnaire and research protocol, and the same well-trained research team responsible for data collection are major strengths of this study.

The primary limitation of this study is that the data is based on self-reported assessment. However, the self-reported measures of body weight and height have been validated, and other studies have revealed high correlations between self-reported and laboratory measurements of BMI, making it suitable for epidemiological studies to identify excessive body weight in children and adolescents [36]. Although every attempt was made to minimize bias, the self-reported measures applied in this study are subject to recall and social desirability bias, which may have affected the responses. The cross-sectional design of this study does not allow us to interpret the results on the relationship between responses and explanatory variables causally. However, previous longitudinal studies point to the beneficial effects of additional school physical activity on reducing obesity in school-age children [80, 81].

## Conclusions

The results of this study pointed out the rising trends of excessive body weight (obesity and overweight), especially in adolescents from low-SES families. Additionally, significantly higher odds of obesity in 11- and 13-year-old adolescents from low-SES families, compared with their peers aged 15, indicated an expectable rise in obesity in older low-SES adolescents in the near future. It seems that association energy expenditure behaviours (participation in individual or team sports, regular weekly vigorous PA, and daily moderate-to-vigorous PA) with weight status in adolescents are stronger than is the case for unhealthy eating habits (daily consumption of sweets or occasional meals at fast food restaurants). We highlight the importance of prevention and the need for more effective strategies/programmes to prevent excessive body weight in children and adolescents with a low-SES background.

## Data Availability

The datasets analysed during the current study are not publicly available because of the rules for funded projects but are available from the corresponding author ES upon reasonable request.

## Abbreviations

BMI: Body Mass Index
CI: Confidence interval
FA: Family affluence
HBSC: Health Behaviour in School-aged Children
ICC: Intra class correlation
MVPA: Moderate-to-vigorous physical activity
OR: Odds ratio
PA: Physical activity
PC: Computer use
Ref.: Reference group
SES: Socioeconomic status
SPSS: Statistical Package for the Social Sciences
ST: Screen time
TV: Television viewing
WHO: World Health Organization
WVPA: Weekly vigorous physical activity
